# Lie to my face: An electromyography approach to the study of deceptive behavior

**DOI:** 10.1002/brb3.2386

**Published:** 2021-10-22

**Authors:** Anastasia Shuster, Lilah Inzelberg, Ori Ossmy, Liz Izakson, Yael Hanein, Dino J. Levy

**Affiliations:** ^1^ Sagol School of Neuroscience Tel Aviv University Tel Aviv Israel; ^2^ Coller School of Management Tel Aviv University Tel Aviv Israel; ^3^ Center for Nanoscience and Nanotechnology Tel Aviv University Tel Aviv Israel; ^4^ Department of Psychology and Center of Neural Science New York University New York City New York USA; ^5^ School of Electrical Engineering Tel Aviv University Tel Aviv Israel

**Keywords:** cognition, electrophysiology, experimental psychology, psychology

## Abstract

**Background:**

Deception is present in all walks of life, from social interactions to matters of homeland security. Nevertheless, reliable indicators of deceptive behavior in real‐life scenarios remain elusive.

**Methods:**

By integrating electrophysiological and communicative approaches, we demonstrate a new and objective detection approach to identify participant‐specific indicators of deceptive behavior in an interactive scenario of a two‐person deception task. We recorded participants' facial muscle activity using novel dry screen‐printed electrode arrays and applied machine‐learning algorithms to identify lies based on brief facial responses.

**Results:**

With an average accuracy of 73%, we identified two groups of participants: Those who revealed their lies by activating their cheek muscles and those who activated their eyebrows. We found that the participants lied more often with time, with some switching their telltale muscle groups. Moreover, while the automated classifier, reported here, outperformed untrained human detectors, their performance was correlated, suggesting reliance on shared features.

**Conclusions:**

Our findings demonstrate the feasibility of using wearable electrode arrays in detecting human lies in a social setting and set the stage for future research on individual differences in deception expression.

## Graphical abstract

What classifies lying from truth‐telling behavior? The current study adopts a physiological‐social approach, combining state‐of‐the‐art techniques from electrical and neuro‐engineering (nanotechnology‐based skin printed electrodes for measuring electromyography), computer science (machine‐learning algorithms), and cognitive psychology (deception task) to automatically and objectively characterize different deceptive behaviors based on “giveaway” signs of facial expressions.

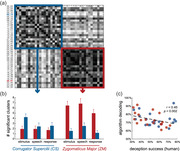

## 1 INTRODUCTION

Deception, “an act that is intended to foster in another person a belief or understanding that the deceiver considers false,” occurs in all walks of life—from telling your colleague you like their new haircut to large‐scale frauds. Although it affects a wide range of fields such as finance (e.g., protection against fraud), business (e.g., gauging a negotiator's credibility), and security (e.g., in border protection), it is still an open question as to how it can be detected. Humans’ ability to identify deceit is poor, with performance around chance (Bond & DePaulo, 2006). Trained expert detectors, such as law enforcement personnel, perform only slightly better on average (Bond & DePaulo, 2006; Ekman & O'Sullivan, 1991), mainly due to their self‐confidence (Vrij, 2008) or bias toward perceiving deception (Meissner & Kassin, 2002). However, recent studies showed that these average improvements in experts’ detection performance reflect nothing more than chance variation (Aamodt & Custer, 2006; Bond & DePaulo, 2008). Thus, what people subjectively think is indicative of deception cannot be reliably used to distinguish lying from truth‐telling (Frank et al., 2008).

For more than half a century, researchers applied a *physiological approach* to deception detection by looking for physiological indicators of lying behavior (see Podlesny & Raskin, 1977 for review). The basic assumption is that lying leads to psychological arousal, which leads to measurable physiological arousal (Kleinmuntz & Szucko, 1982; Lykken, 1979). An impressive host of responses correspond to this arousal, such as changes in blood pressure, pulse rate, respiration, and galvanic skin response (Grubin & Madsen, 2005). In a polygraph test, for example, an investigator measures an examinee's responses to a series of questions (Committee to Review the Scientific Evidence on the Polygraph, 2003; Horvath & Reid, 1971). Indeed, the polygraph is the most popular instrument for lie detection with a high reported accuracy of ∼86% (Committee to Review the Scientific Evidence on the Polygraph, 2003). However, even though the polygraph test relies on objective physiological responses, the collection and interpretation of its data are highly subjective. For example, the questions are not similar across all tests, and different investigators use different values to indicate a lie (Steinbrook, 1992). Moreover, the physiological responses can be reduced or feigned to mislead the investigator (Ben‐Shakhar & Dolev, 1996; Bersh, 1969; Saxe et al., 1985), or they can be elicited by emotional processes, such as stress and anger that are not necessarily related to deception (Steinbrook, 1992).

Another traditional line of research takes a *communicative approach* to deception detection by focusing on social indicators of deception, such as facial expressions. Facial features are salient cues for social attribution. For example, less than 100 ms are sufficient to judge whether a static face is trustworthy or threatening (Todorov et al., 2015). Dynamic facial expressions are a central component of emotional expression upon which we rely to convey our feelings and intentions and infer those of others. It was Darwin who first noted that some emotions are too great to be fully feigned or concealed, and that some facial expressions might “leak,” revealing true feelings (Darwin, 1872). This concept was adopted into a systematic lie detection approach by using the Facial Action Coding System (FACS; Ekman & Friesen, 1976). FACS consists of action units (AUs) that represent observed facial movements and their combinations. The theory behind the use of FACS to identify lies posits that deception manifests itself through involuntary micro‐expressions, that are transient (40–60 ms) and incongruent to the emotion the person is trying to convey (Ekman & O'Sullivan, 2006). Nevertheless, FACS is susceptive to biases and inaccuracies that are not necessarily related to deception (Barrett et al., 2019; Wolf, 2015).

**FIGURE 1:**
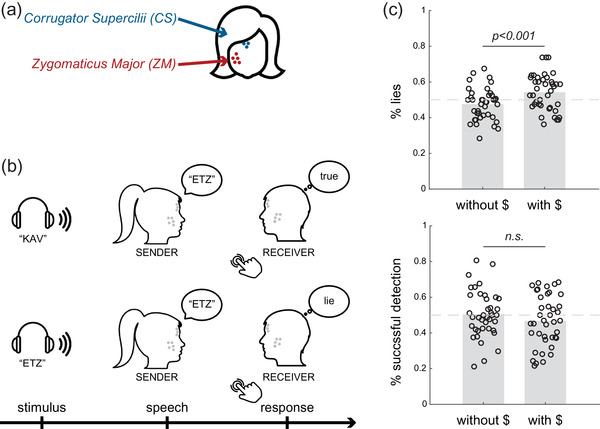
**Setup, task procedure, and behavioral results**. The participants completed a two‐person deception task, while their facial muscle activity was recorded using facial surface electromyography. (a) An eight‐electrode array was placed on each participant's face, with five electrodes recording from the *zygomaticus major* muscle (cheek region) and 3 from the *corrugator supercilia* muscle (eyebrows regions). (b) The participants took turns acting as Sender and Receiver. The Sender would hear a message via earphones (either “KAV” or “ETZ”; stimulus event), then would either repeat the word (Truth) or utter the other word (Lie; speech event). Then, the Receiver would indicate via key press whether they believed the Sender (Truth) or not (Lie). Depicted are two examples of trials: Lie‐Truth (top panel) and Truth‐Lie (bottom panel). (c) The participants lied in approximately half of the trials, and the frequency of lying increased between the two stages of the experiment (top panel). The Receivers’ detection of the Senders’ lying was at chance level, and time and monetary incentives did not change their performance (bottom panel)

Facial surface electromyography (sEMG) is a reliable technology often used to quantify facial expressions by recording the electrical activity of muscles located (that is, recording the electrical activity generated by facial muscles close to the skin; Schumann et al., 2010). sEMG can indicate facial muscle activation even when this activation is too subtle to be noticed visually by humans (Petty & Cacioppo, 1986). Therefore, sEMG is an appropriate choice for objective deception detection (Samuel et al., 2019), and in cases where facial impressions are too subtle for humans to detect. However, assessment of facial expressions through muscle activation patterns poses challenges for studies of deception. The recording technology must have high resolution and muscle specificity (Hug & Tucker, 2018; Van Boxtel, 2010), while keeping the participants comfortable. Indeed, contemporary facial sEMG devices are cumbersome, unstable, prone to noise, record only for a limited time, rely on expert placement, and produce signals with low spatial resolution (Hug & Tucker, 2018; Wolf, 2015). Recently developed dry screen‐printed electrode arrays (Figure 1a and Figure S1; Bareket et al., 2016) offer a new and improved alternative that overcomes these shortcomings.

The current study is an attempt to combine electrophysiological and communicative approaches to examine the ability to achieve sensitive deception detection from facial sEMG and machine‐learning algorithms. The unique electrodes we used are soft, conform to the shape of the skin, can record for hours in a stable manner, with high signal‐to‐noise ratio, and can be used in a psychologically ecological manner (Inzelberg et al., 2020; Inzelberg, Pur, et al., 2018; Inzelberg, Rand, et al., 2018). Moreover, we used supervised machine learning (support vector machine classification and unsupervised peak‐density clustering) to identify “give‐away” indicators of deceptive behavior. Our primary goal was to test the feasibility of using brief facial sEMG signals as an objective indicator of lying during face‐to‐face interactions. Specifically, we examined spatial factors (i.e., *zygomaticus major* and *corrugator supercilia* muscles) and temporal factors (i.e., signal timing, delivery timing, or the Receivers’ response latency) of the sEMG signal that could explain individual differences in deception. In the first stage of the experiment, the participants were not compensated for successful deception nor detection. In the second stage of the experiment, we introduced monetary incentives to examine how lying behavior (and its accompanying facial expressions) changed as time passed and circumstances changed.

## 2 METHODS

### 2.1 Participants

Forty‐eight participants were enrolled in the study (35 females; *M*_age _= 23.67, range: 18–30). The participants gave informed written consent before participating in the study, in accordance with relevant guidelines and regulations under approval from the Institutional Ethics Committee Review Board at Tel Aviv University. Six participants were excluded due to technical issues with the recording equipment, and another two for never lying. All trials from those participants were excluded and analyses were conducted on the resulting 40 participants. The participants were compensated for their time and success in undetected lying. Each participant contributed *M* = 158.32 trials (*SD* = 6.49). For all the participants, we carried out at least 152 trials, except for one participant (id: 12_23) who had only 40 out of 80 trials when monetary incentives were not presented. We excluded trials due to technical issues (i.e., we were unable to identify the onset of the participant's speech, the content of what they said or the EMG signal was too noisy).

### 2.2 Procedure

Pairs of the participants sat in a relaxed upright position facing each other and were equipped with earphones and microphones that recorded and saved each trial. Their skin was mildly cleaned and exfoliated (Everi, SpesMedica) prior to electrode array placement. The participants were asked to smile and frown to locate the *zygomaticus major* (ZM) and *corrugator supercilii* (CS) regions. Accordingly, customized screen‐printed facial electrode arrays (eight electrodes; 5 mm in diameter) were adhered to the participants’ right side of the face. Five electrodes (0–4) were located at the cheek region and three (5–7) at the region of the eyebrows, above the ZM and CS muscles, respectively, in all the participants, as described in detail in Inzelberg et al. (Figure S1; Inzelberg, Rand, et al., 2018). The electrode arrays were connected to two amplifiers (RHD200; Intan technologies) using a customized zero‐insertion–force (ZIF) connector. A disposable commercial ground plate electrode (019—409100; NATUS) was positioned on the bony prominence of the seventh vertebra (C7). Both participants performed a calibration phase (in response to a cued video) of three voluntary smiles followed by three contractions of the eyebrows with a neutral expression in between. Each expression was performed for 3 s with a 3‐s gap in between (Figure S1). The participants were allowed to move their heads freely during the recording and were instructed to look at their partners throughout the experiment.

### 2.3 Task

#### 2.3.1 Stage 1 (no monetary incentives)

The participants took turns acting as *Sender* and *Receiver* in a two‐person deception task (Figure 1b). During each trial, the Sender (and only the Sender) heard one of two possible words (the Hebrew words “KAV” or “ETZ,” “line” or “tree,” randomly) through the earphone. We chose these words as they are both comprised of a single syllable. The Sender was then free to either repeat the word (i.e., convey a truthful message to the Receiver), or say the other word (a deceitful message). After delivering the message, the Receiver indicated, via keyboard press, her subjective evaluation of whether the Sender told the truth (i.e., the word that the Sender heard is congruent with what the Sender said), or lied (heard a different word than the one said). We did not give the Receivers any instructions about the response speed. To conclude, each trial unfolded in one of four manners: The Sender lied but the Receiver thought the Sender was telling the truth (Lie‐Truth, L‐T); the Sender lied and the Receiver did not believe it (Lie‐Lie, L‐L); the Sender told the truth and the Receiver believed him (Truth‐Truth, T‐T); the Sender told the truth but the Receiver thought it was a lie (Truth‐Lie, T‐L). Whether or not the Sender actually told a lie was not revealed to the Receiver, and also the Receiver's responses were not revealed to the Sender, to avoid learning on either side. Each participant completed two blocks of 40 trials each as the Sender, and another two blocks as the Receiver. The participants alternated roles between blocks. We encouraged the Senders to vary their responses across the experiment (not to lie or tell the truth all the time).

#### 2.3.2 Stage 2 (with monetary incentives)

After completion of 160 trials (80 each), we introduced monetary incentives to the task and the participants performed an additional 160 trials (80 per participant). The design was identical to stage 1 but the Sender was monetarily incentivized to successfully mislead the Receiver (i.e., make them believe they told the truth when they in fact lied and vice versa), whereas the Receiver was compensated for successful detection of both lies and truths. The Sender's payoff structure was as follows: six points for successful lying (L‐T), four points for misleading truth‐telling (T‐L), and two points for any other case (T‐T and L‐L). The Receiver's payoffs were four points for successful detection (T‐T and L‐L), and zero points otherwise. Each point in the task was worth 0.2 NIS (1 NIS ≈ 0.3 USD). Using a speech‐processing algorithm, we assessed the participants’ ongoing performance and computed their winnings by the end of the experiment.

### 2.4 Behavior analysis

We computed each participants’ rate of lying, truth‐telling, and successfully misleading as Senders, as well as their lie detection performance as Receivers (proportion of L‐L from all lies). To test how differences in reaction times relate to deception and detection, we examined two types of reaction times (RT): Sender RTs, measured as the time from stimulus onset to speech onset, and Receiver RTs, measured from (Sender's) speech onset to (Receiver's) response onset. To identify speech onsets, we used the audio recording of each trial. To examine how repetition (i.e., time in the experiment) and monetary incentives affected lying, we conducted a logistic regression of lying on each trial as a function of trial number and a dummy variable of monetary incentives, clustering the errors per participant.

### 2.5 sEMG preprocessing

Data analysis was performed using MATLAB R2019a. sEMG data were recorded with a sampling rate of 2000 samples/s. We computed 28 permutations of differential sEMG data. Data were then filtered using a 50 Hz notch filter and a band‐pass 4 order Butterworth filter in the frequency range of 5–500 Hz. After visually inspecting the sEMG signal recorded during a calibration phase (Figure S1), for each participant, we chose two differential sEMG channels if they clearly depicted frowning from the CS muscle region (electrodes 5–6 or 6–7), and smiling from the *zygomaticus* muscles region (e.g., electrodes 2–3, 2–4, etc.). We then used these two differential channels for the following analysis process.

We smoothed the sEMG signal in each epoch by extracting its peak envelope (determined by the spline interpolation over local maxima in a 50‐ms time window). Then, the continuous differential sEMG envelope was split into the different trials based on the online triggers that were sent by a custom‐built software during the experiment. For each trial, we cut the trial to three 1 s event‐related epochs: (1) 0–1 s relative to trial onset (the epoch containing the *Stimulus* played over the earphones); (2) 0–1 s relative to Sender's *Speech* onset; and (3) −1 to 0 s relative to Receiver's *Response*. The epochs could overlap (e.g., when the *Receiver* responded in less than 1 s after the Sender started to speak). The sEMG epochs were used as input to the classifier. To summarize, preprocessing consisted of filtering, channel differentiation, smoothing, segmenting into trials, and segmenting into event‐related epochs (*Stimulus*, *Speech*, *Response*; see Figure S2).

### 2.6 Classification procedure and deception detection matrices

**FIGURE 2:**
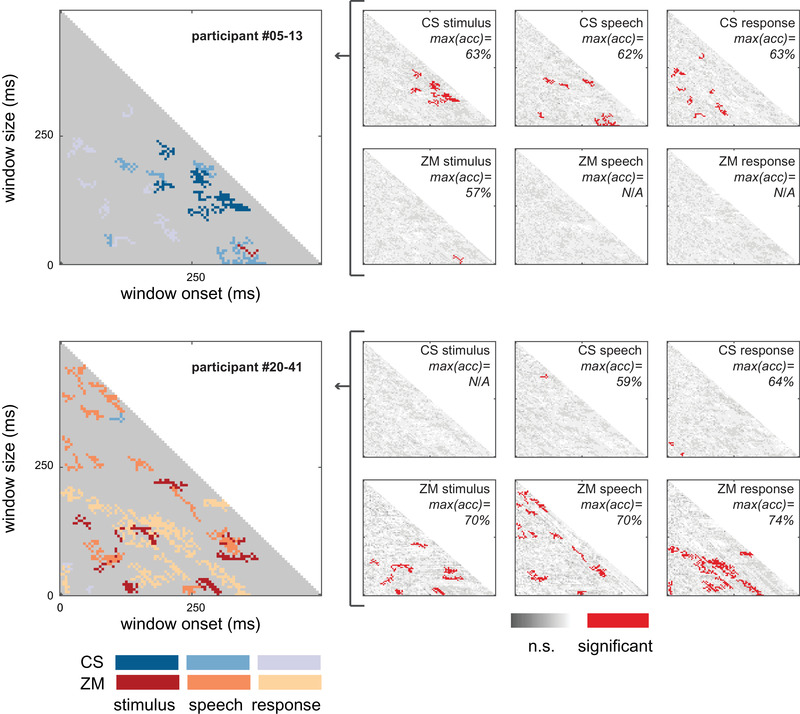
**Deception Detection Matrices (DDMs) of participants #05–13 and #20–41**. Right panels: Each matrix element was derived from 1 s event‐related epoch from either the ZM or CR facial muscle regions, during stimulus, speech, or response. Red indicates a successful lie detection. Data were calculated for bins varying in duration (*y*‐axes) and sampled starting at varying time‐points relative to the event onset (*x*‐axes). Big panels: For each participant, the six DDMs were aggregated into a single matrix. Color coding was used to indicate muscle region and trigger (i.e., stimulus, speech, or response)

To find the specific features that are relevant for deception within the Sender's behavior, we discriminated “truth” trials (both T‐T and T‐L) and “lie” trials (L‐L and L‐T) using MATLAB implementation of a support vector machine classifier (SVM; Chang & Lin, 2011; software available at http://www.csie.ntu.edu.tw/~cjlin/libsvm) and least‐squares as a cost function. To take advantage of the high temporal resolution of the sEMG recording, we classified “truth” and “lie” trials in smaller time windows by making 10 ms increments in the window onset (i.e., classifying “truth” and “lie” from time 0−1000 ms in the first step, then from time 10–1000 ms in the following step, then 20–1000 ms, and so on until the final time bin 990−1000 ms), and changing the size of the window in 10 ms increments (i.e., classifying from time 0–10 ms in the first step, then from time 0–20 ms in the following, then 0–30 ms, and so on until 0–1000 ms). We conducted a classification for each of all the possible pairs of window onsets and window sizes. This procedure, similar to previous work in Neuroscience (Ossmy et al., 2015; Reaz et al., 2006) resulted in a deception detection matrix (DDM)—an m‐by‐m matrix where the (ith, jth) cell of the matrix is the accuracy of classifying “truth” and “lie” trials for a window onset i and window size j, and *m* is the total number of 10 ms bins (100 bins) in each event‐related epoch. The DDM has values only in the lower triangular region because the window size is determined by the onset of the window (see Figure 2).

For each time window, the following classification procedure was implemented (Figure S2): First, we applied principal component analysis on the sEMG signal (Bosco, 2010) (in the specific time window only) across all 80 trials. Then, we provided the Support Vector Machine (SVM) with (1) data items—two components representing the pattern of sEMG activity in a trial (two components explained more than 95% of the variance across trials in all the participants); and (2) labels—indication whether the participant lied on each trial (“truth” or “lie”). To test the classification accuracy, we randomly chose data from one trial from each label as a test set, and the SVM classifier was trained on the remaining two datasets (trials in which the participant lied and trials in which the participant told the truth). Following training, we assessed the classification performance on the test set (“leave‐one‐trial‐out”). To verify that the classifier did not learn any property of the tested exemplar, which might otherwise appear in the training set, we performed cross‐validation by using a leave‐one‐out procedure—the average performance level across 1000 iterations (different test set in each iteration) was assigned to each of the time onset‐size window of the DDM.

We aimed to identify portions of the sEMG signal that could significantly distinguish lying from truth‐telling. Thus, we generated for each participant DDM maps of classification accuracy based on the two predefined regions of the face: (1) the *zygomaticus major*; and (2) *corrugator supercilii* muscles, and the three events in a trial: (1) *Stimulus*—1 s after stimulus onset; (2) *Speech*—1 s after speech onset; (3) *Response*—1 s leading up to the Receiver's response, for a total of six DDMs per participant.

To assess the statistical significance of the classification performance level, we generated 1000 “shuffle” DDMs (per event, region, and participant) in which the classification was based on shuffled labels as input to the classifier. We used a data‐driven approach to detect time windows with significant classification. Subsequently, we performed a nonparametric cluster analysis on the accuracy level in both the shuffled and real DDMs and assessed statistical significance using a well‐established clustered‐based statistical procedure that accounts for multiple comparisons (Maris & Oostenveld, 2007). The procedure was as follows (Figure S2): for each participant, we (1) defined a statistical threshold as the 95th‐percentile accuracy level across all shuffled‐DDMs; (2) selected all the bins in each DDM (real and shuffled) that exceed that threshold, and clustered them on the basis of temporal adjacency (minimal cluster size = 2); (3) took the sum of the accuracy values within a cluster per DDM as a cluster‐level statistic; (4) took each shuffled‐DDM's best‐performing cluster (largest of the cluster‐level statistics), resulting in a 1000 accuracy values; (5) finally, a cluster from each of the six real DDMs was considered significant if its performance exceeded those 1000 values. The number of shuffled DDMs sets an upper bound on our significance level at *p* < .001.

The entire analysis pipeline that we used conforms to guidelines from previous work that used machine learning to analyze behavioral and neural data in relatively small samples (Abou Elassad et al., 2020; Lakertz et al., 2021; Ossmy & Adolph, 2020; Ossmy et al., 2014, 2015). We separately implemented the analysis pipeline on the data from stage 1 of the experiment (no monetary incentives) and stage 2 (with monetary incentives).

### 2.7 Multi‐participant analysis: Grouping

To identify potential subtypes of liars in our sample, we performed a second‐level, multi‐participant analysis. For each participant, we counted the number of significant clusters in each of the participant's six DDMs (2 muscle regions × 3 events). This procedure resulted in a six‐cell vector per participant that can be viewed as encapsulated information about the participant's pattern of deception.

We then compared the participants based on the similarity of their pattern of deception. The deception pattern of each participant was represented as a vector that includes the number of clusters in each one of the six DDMs. Similarity was measured by the six‐dimensional Euclidean distance between each pair of vectors. This procedure yielded a 40×40 matrix in which cell i,j is the “distance” between participant i and participant j,based on the number of clusters in their DDMs. If the distance between the participants is high, the similarity between them is low and vice versa.

Next, we followed the “fast search and find of density peaks” clustering method (Rodriguez & Laio, 2014) to group the participants based on their deception (Figure S3). With this method, we first identified the participants that are “group prototypes” and then classified the rest of the participants according to the “group prototype” to whom they were most similar. Similar to Rodriguez and Laio (2014), a group prototype participant was characterized by: (1) *high density* in the similarity matrix (i.e., many participants were relatively similar to them); (2) *large distances* from the participants with higher densities (i.e., relatively low similarity to the other participants with high densities).

Finally, each participant was assigned to the “group prototype” to whom they were most similar. Most importantly, in this procedure, the number of groups is derived from the data and it is not predefined as in other, more common methods, such as k‐means clustering (Hartigan & Wong, 1979).

After clustering, we looked for group characteristics. We used paired *t*‐tests to test for each group's difference between an overall number of ZM clusters and an overall number of CS clusters (to test for spatial characteristic), and a repeated one‐way analysis of variance (ANOVA) between an overall number of Stimulus clusters, an overall number of Speech clusters, and an overall number of Response clusters (temporal characteristic).

### 2.8 Multi‐participant analysis: Correlation with behavior

We examined whether our classifier's ability to detect lies is similar to the human ability of the Receiver to detect lies. As an overall measure of the machine detection performance per participant, we took each participant's maximal classification accuracy across all DDMs. As a behavioral measure, we took the Sender's success in misleading the Receiver—the proportion of trials in which the participant lied, and the Receiver responded “truth” (L‐T trials). Using a two‐tailed paired *t*‐test, we compared the classification detection performance with that of the Receiver's. Using Spearman correlation, we examined the correspondence between the two measures.

## 3 RESULTS

The experimental setup consisted of a two‐person task, in which participants were asked to trick each other (see Figure 1b). While sitting face‐to‐face, they took turns being the Sender or the Receiver. The Senders heard a signal in their earphone, unbeknown to the Receivers, and delivered a message to the Receivers, which could either be truthful (congruent with the signal they heard) or deceitful (incongruent). The Receivers were instructed to tell whether the message was true or false.

### 3.1 Behavioral results

The participants lied on 50.96% of the trials on average (*SD *= 7.77) (Figure 1c). The Receivers’ detection rates varied between the participants, from 22% to 72.83% of the Senders’ lies (*M *= 48.48%; *SD *= 11.84), but were not different than chance at the group level (*t*(39) = −0.81, *p* = .42, one‐sample *t*‐test) (see Figure 1c).

The Senders’ reaction times—the time interval from stimulus onset to speech onset—did not differ whether the Sender told the truth or lied (*M*_truth _= 1.89 s, *SD*_truth _= .84*; M*_lie _= 1.87 s, *SD*_lie _= .79; *t*(39) = 0.83, *p* = .40). Similarly, the Receivers’ reaction times—the time interval from speech onset to response onset—did not differ between trials when the Sender lied and when the Sender told the truth (*M*_truth _= 4.35 s, *SD*_truth _= 1.37*; M*_lie _= 4.43 s, *SD*_lie _= 1.54; *t*(39) = −0.67, *p* = .50).

To assess whether lying behavior changed throughout the experiment, we conducted a logistic regression analysis of lying as a function of time (trial number) and the existence of monetary incentives. Although incentives did not affect the probability of lying (coefficient = 0.05, *p* = .55), the passage of time increased it (coefficient = 0.002, *p* = .012). We, therefore, proceeded to separately examine behavior without and with monetary incentives. We found a near‐significant effect that participants who tended to lie more in the first stage also lied more in the second stage (*r*(38) = *r*: 0.29, *p* = .06; bend correlation; *r*(38) = 0.32, *p* < .04; Pearson correlation). We also found the participants lied more in the second stage (*M*_stage2_ = 54.26%, *SD *= 10.10; *M*_stage1_ = 47.55%, *SD *= 8.91, *t*(39) = 3.82, *p* < .001; paired *t*‐test). Conversely, although detection rates in the two stages of the experiment were correlated (*r*(38) = 0.45, *p *< .001), the Receivers did not improve with time and the introduction of monetary incentives (*M*_stage2_ = 46.76%, *SD *= 14.29; *M*_stage1_ = 50.44%, *SD *= 13.12, *t*(39) = −1.62, *p* = .11; paired *t*‐test).

### 3.2 Classifying lies in individual participants

To find the specific features associated with deception behavior of the Senders, we discriminated “truth” trials (T‐T and T‐L) and “lie” trials (L‐L and L‐T) using a support vector machine classifier (SVM; Chang & Lin, 2011), and least squares as a cost function. Figure 2 depicts the DDMs calculated from the sEMG data of two exemplar participants (#05–13 and #20–41). We calculated 1 s event‐related epochs from two facial regions (ZM and CR) during stimulus, speech, and response events. Data were divided into bins varying in duration (Figure 2, *y*‐axes) and sampled starting at varying time‐points relative to the event onset (Figure 2, *x*‐axes). These bins were used as input for an SVM classification algorithm (Figure 2, right panels), that classified “truth” and “lie” trials. After correcting for multiple comparisons, significant clusters were aggregated across each participants’ six DDMs (left‐hand side). As clearly evident from the big panels in Figure 2, participant #05–13 had significant clusters mostly from the CS muscle (blue tones), while participant #20–41 had significant clusters mostly from the ZM muscle (red tones). In both cases, lie detection was successfully achieved. Using this approach, we successfully detected lies in all (40) the participants (Figure S2). This is the first evidence for sEMG‐based micro‐expressions that reveal lies in face‐to‐face conditions.

Importantly, we also noticed that the participants changed their deceptive behavior between the two stages of the experiment (with and without monetary incentive). Accordingly, we analyzed the sEMG signal from the first stage of the experiment (without monetary incentives) and the second stage (with monetary incentives) separately. In the first stage, our maximal success in classifying whether a specific participant lied was *M *= 72.97%, *SD *= 7.34 (averaged across the participants). Classification was slightly better using data from the second stage of the experiment, after presenting monetary incentives (*M *= 73.65%, *SD *= 8.00), but the increase was not significant (*t*(39) = 0.95, *p* < .34; paired *t*‐test).

Per participant, the classifier identified an average of 16.10 clusters (±9.95) that significantly distinguish lies from truths (summed over all 6 DDMs; see Tables S1 and S2 for full list). We identified more clusters in trials from the first stage (*M *= 19.00, *SD = *11.53) than in trials from the second stage (*M *= 13.20, *SD = *7.09; *t*(39) = 3.43, *p* < .001; paired *t*‐test). The number of clusters per DDM and per subject varied (see Tables S1 and S2). Some DDMs had no significant clusters, while others had up to 44 clusters. We found high inter‐individual variability, such that none of the muscle regions or events were significantly dominant across all the participants (regardless of the experiment stage, *ps* > .23; paired *t*‐test comparing the two muscle regions across the participants). Some participants had most of the clusters accumulated within one or two DDMs, while others had clusters spread across all DDMs.

### 3.3 Grouping participants

**FIGURE 3:**
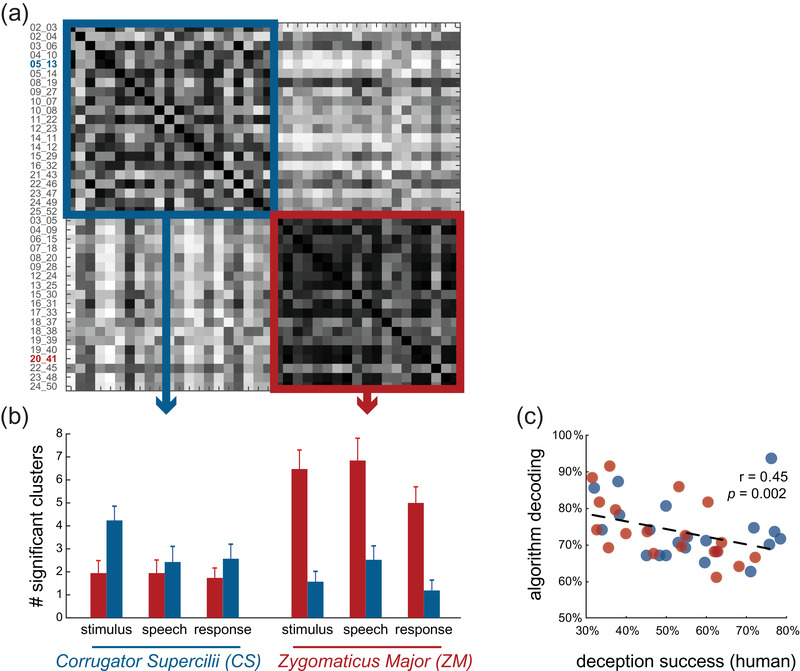
**Multi‐subject result reveals two types of liars**. (a) Results of a similarity analysis between the participants based on classification performance in each of the six DDMs (2 facial muscles × 3 trial events) based on surface electromyography (sEMG) data from the second stage of the experiment (with monetary incentives). A clustering algorithm identified two distinct groups of the participants based on similarity (blue and red squares). The IDs of the two exemplary participants from Figure 2 are highlighted. (b) The number of significant clusters in each of the six DDMs, averaged across the participants of each group. The differences suggest that the blue group's classification mostly relied on data from the eyebrow muscle (CS), and the red group has more classification success using data from the cheek muscle (ZM). (c) The ability of the classification algorithm to detect a participant's deception (measured as maximal classification accuracy) is negatively correlated with the ability of that participant to deceive their human counterpart (Receiver). Each circle represents a participant, colored based on their group belonging (as depicted in (a))

The high individual differences in the number of clusters within DDMs provide an opportunity to test whether there are *types of liars*, differing in their “give‐away” indicators—either spatially or temporally. Specifically, we examined whether the identity of the facial muscle contributed more to detection ability (ZM or CS) or the event (*Stimulus*—when the participants heard the stimulus, *Speech*—when the participants delivered the message, *Response*—when the participants were awaiting a response). Using density‐peak clustering (see Section 2 and Figure S3), in the second half of the experiment, when monetary incentives were presented, we identified two groups—G1 with 21 participants, and G2 with the remaining 19 participants (Figure 3a). The average number of clusters per DDM within each group revealed that the region the electrodes were recording from is a dominant feature in grouping the participants. While G1 is characterized by a larger number of clusters in DDMs of CS data (*t*(20) = 2.95, *p* < .01), G2 has more clusters of successful classification using the ZM data (Figure 3b; *t*(18) = 6.35, *p* < .001). The number of clusters in the temporal events did not differ; therefore, the temporal events did not characterize either group and were not a dominant factor in clustering (*F*(2.19) = 1.11, *p* = .35 for G1, *F*(2.17) = 3.19, *p*s = .07 for G2).

We similarly found two groups using the data from the first stage of the experiment, when monetary incentives were not yet introduced. G1 in this case had 21 participants and G2 had the remaining 19, although not the same 21 (19) participants comprised G1 (G2). Again, the dominant factor in clustering was the muscle region (G1: *t*(20) = 2.69, *p* < .02; G2: *t*(18) = 6.67, *p* < .001), and not the temporal events (G1: *F*(2.19) = 1.7, *p* = .21; G2: *F*(217) = 0.91, *p* = .41)

### 3.4 Behavior and sEMG correlation

To relate our classifiers’ detection accuracy to the Senders’ ability to lie successfully (i.e., deceive the Receivers), we computed each participant's overall machine‐detection success (maximal classification accuracy; see Section 2). We then compared the classifier's performance with the Senders’ successful‐lying measure—the percentage of times the Sender told a lie and was not caught by the Receiver (L‐T trials). We found that the classifier was significantly better at detection than humans (i.e., Receivers) both in the first and second stages of the experiment (*t*(39) = −6.8, *p* < .001 and *t*(39) = −8.4, *p* < .001, respectively), and that successful lying behaviorally is negatively correlated with classification success (Figure 3c), again in both stages of the experiment (*r*(38) = −0.45, *p *< .001; *r*(38) = −0.42, *p *< .001; bend correlation).

## 4 DISCUSSION

We adopted a novel physiological‐communicative approach to deceptive behavior, combining a state‐of‐the‐art recording technique and advanced software algorithms (supervised classification and unsupervised clustering). We used a paradigm that requires the participants to choose between two actions where one reflects the truth and the other reflects a lie and used the participants’ responses as a *model system* for investigating specific indicators of deception. Unlike previous studies in this field, our approach comes closer to real‐world scenarios by confronting the participants with the receivers and testing deception in a social, interactive context, and their choices had very real consequences on their monetary payoffs, adding ecological validity. Moreover, our presented approach does not require high‐resolution video recording and analysis. These demand high computational time, the ability to track subject movements, the need for a frontal view, high frame rate, and a well‐lit environment. Currently, computer vision approaches showed limited success in detecting leaked expressions, particularly in face‐to‐face situations (Merghani et al., 2018). On the other hand, we successfully detected lies in all the participants and did so significantly better than untrained human detectors. Not only was facial muscle activity sufficient to detect human lies, but we also found that humans differed in their “give‐away” indicators. Some revealed their lies by moving their cheeks (the ZM muscle), whereas others expressed lies by moving their eyebrows (CS). Interestingly, individuals who were able to successfully deceive their human counterparts were also poorly detected by the machine‐learning algorithm.

### 4.1 Not all liars are created equal: Indicators of deception are not universal

Our individual‐level, within‐participant analyses were designed to identify key factors that contribute to detecting lies of specific individuals—both temporal events (when people heard the stimulus, delivered the message, or awaited a response) and facial muscles (ZM and CS). The fact that we identified different types of liars goes against the idea that expression of deceit has universal indicators (Barrett, 2006; Ekman, 1999; Frank & Svetieva, 2015), but rather suggests that there are at least two types of “give‐away” indicators of lying. This inter‐individual difference possibly explains the poor performances of existing approaches to lie detection, as both the physiological and communicative approaches rely on a set of predefined indicators, assuming that people share similar indicators to deception. Findings based on our data‐driven, unbiased approach, suggest that this assumption is only partly true. Indeed, the participants did not differ in *when* their indicators of deception appeared; however, they did differ in *where* the indicators were located on the face. This is not to say that we believe that instead of one display of deception there are two—but rather that there is a host of possible manifestations of deception, and we have merely uncovered two of them.

The locations of the deception indicators we identified here suggest inter‐individual differences in how individuals express their emotions. Both ZM and CS muscles have well‐known roles in emotional expression. ZM activity correlates with positive affect and expressions such as smiling, whereas CS activity correlates with negative affect and expressions such as frowning (Dimberg, 1988; Larsen et al., 2003; Schwartz et al., 1979). For example, previous studies demonstrated that the participants frown more when reading about the immoral actions of other people (t Hart et al., 2018), and that CS can help track responses to affectively loaded language (Foroni & Semin, 2009, 2013; Glenberg et al., 2009; Niedenthal et al., 2019). Future work could pinpoint the mapping between facial muscle activity during emotional expressions and facial muscle activity during deception. Furthermore, while previous sEMG work relied on precise electrode placement to accommodate structural diversity, our investigation focused on the general muscle location. This is because the CS (upper part of the face) and ZM (lower part of the face) are relatively far apart. Future work should consider using source separation methodologies to investigate the specific muscles activation role in deception rather than their general region.

The correlation between the Receivers’ success in detecting lies and the algorithm classification accuracy suggests that even though the classifier was a better detector, both the human and the algorithmic detectors relied on similar indicators of deception. Indeed, facial muscle activity is known to play a role in how humans detect lies (Van Bockstaele et al., 2012). These findings support previous work that examined what type of information people most often use to detect real‐life lies (Park et al., 2002). As in the current study, previous questionnaire studies showed that Receivers detect deception mostly by looking at Senders’ nonverbal physical expressions. Moreover, in a few studies (De Turck & Miller, 1990; Fiedler & Walka, 1993), human lie‐detectors (e.g., judges) were trained to look for observable physical expressions when assessing truthfulness. We did not compare the accuracy of the classifier to experts who are already trained in detecting lies. However, other studies showed that experts detect lies 55.74% of the time (Bond & DePaulo, 2006), which is lower than the classification accuracy in the current study (72.97%). Thus, facial sEMG has the potential to help develop improved training regimens to detect deception that focuses on identifying individual differences in small movements of facial muscles.

The idea that facial expressions are the “universal language of emotions” is hotly debated, and we are certainly not the first to challenge the universality or biological hardwiring of facial expression (Jack et al., 2012). However, much of the debate today deals with cultural differences in emotion recognition and expression, while we go a step further and uncover inter‐individual differences *within* a culture. Notwithstanding, applying our framework to participants of different cultures would be both interesting and important.

### 4.2 A lie is a lie is a lie? Deception changes with time

Even within the same individual, lies do not always manifest themselves in the same way, further hindering the universality hypothesis. Behaviorally, the participants in our task lied more often as they got familiarized with the task, amounting to an average increase of 8% between the two stages of the experiment. Yet, the introduction of monetary incentives did not have an effect above and beyond the effect of time. Monetary incentives also did not improve the Receivers’ ability to detect lies, suggesting that detecting lies is not a matter of increased motivation per se. This finding is consistent with previous studies reporting that motivation can actually hinder lie detection (ten Brinke et al., 2016). Importantly, because the order was not counterbalanced, we cannot speak to the effect of monetary incentives on lying without confounding it with the effect of repetition and experience.

As in human lie detection, there was no significant difference in classification accuracy between early and late stages of the task. Nevertheless, we find that the behavioral shift in the Senders’ lying accompanies a shift in facial expression for some participants. In the later stage of the task, their “give‐away” indicators change compared to the earlier stage. In other words, some participants who had ZM as the indicator of deception in trials from the first part of the experiment had CS as the indicator in the second part, and vice versa. These findings also expand previous work that points to people's motivation to lie as one of the factors affecting indicators of deception (DePaulo et al., 2003). Further research must be undertaken to investigate how different aspects of lying relate to facial muscle activity in individuals.

### 4.3 Applications and future directions

Our findings set the stage for the development of a novel autonomous tool for detecting lies by combining recordings of facial muscle activity and machine‐learning algorithms. However, given the individual differences we found, this approach should first be validated by identifying specific indicators in a big, independent and diverse set of examinees (objectively, using machine learning) and classifying their lying and truth‐telling behaviors. Moreover, our task is overly simplistic and does not simulate a realistic situation. The degree to which lies can be detected in a natural environment could potentially be improved by integrating other recent detection technologies that focus on speech (both content and tone of voice; Tao et al., 2019), body language (Wu et al., 2018), and other physiological measures (see review in Burzo et al., 2018). Importantly, detection can be improved not only by adding more data sources, but also by combining other established classifiers (e.g., k‐nearest neighbors, decision tree, Naïve Bayes).

Future studies are needed to establish facial sEMG as a reliable and stable indicator of deception. First, our task should be expanded to scenarios where lies are more substantial, arduous, and more ecological. More substantial lies could yield larger facial expressions that activate a variety of facial muscles more intensely and for longer periods of time. Second, the current study focused on lying. Facial muscle activity should be examined during other types of deception such as omission, evasion, and equivocation. Finally, other measures should be taken in parallel to muscle activity to investigate whether the source of inter‐individual variability in deception comes only from the muscles or a combination with other social and physiological factors.

In addition to lie detection, our task and machine‐learning techniques could be used to assess inter‐individual variability in the expression of other emotions and how they relate to cognitive skills (Bovard et al., 2019), opening new avenues for research and investigation. Previous studies already harnessed deception tasks for testing the role of cognitive appraisal in triggering specific emotions (Khan et al., 2009). Yet, due to conceptual and methodological limitations, significant gaps exist regarding the individual's physiological reactions that underlie the evoked emotions and how those relate to cognition. The tool used in the current study may be used to fill this gap.

## 5 CONCLUSIONS

What indicates deceptive behavior? By bringing together techniques from cognitive psychology (an interactive deception task), electrical and neuro‐engineering (electromyography), and computer science (machine‐learning algorithms), we show that brief facial muscle activity can significantly indicate lying versus truth‐telling. Individuals differed in the muscles that “gave away” their lies, suggesting that the expression of deception is not universal. As time passed and the participants lied more often, the telltale muscles changed, so similar lies could be expressed differently in the facial muscles manifestations.

## CONFLICT OF INTEREST

Lilah Inzelberg and Yael Hanein declare a financial interest in X‐trodes Ltd, which holds the licensing rights of the screen‐printed electrode technology cited in this paper. Both authors have no other relevant financial involvement with any organization or entity with a financial interest in or financial conflict with the subject matter or materials discussed in the manuscript apart from those disclosed. The other authors declare no conflict of interest.

### PEER REVIEW

The peer review history for this article is available at https://publons.com/publon/10.1002/brb3.2386

## Supporting information

Supporting Information

Supporting Information

## Data Availability

The custom‐built software that we used to run the experiment can be downloaded at https://anonymous.4open.science/r/c352cbe5‐6f26‐4d4c‐ad12‐d8f3bf50c4c2/. Aggregated data is provided in Supplemental Materials. Raw data that support the findings of this study are available upon request from the corresponding author. Raw data are not publicly available due to ethical restrictions.
