# Supplementary material for: Lie to my face: An electromyography approach to the study of deceptive behavior
Source: Brain Behav. 2021 Oct 22;11(12):e2386. doi: 10.1002/brb3.2386 (PMC8671780; doi:10.1002/brb3.2386)

Table S1. Classification results— Stage 1: without monetary incentives. Each participants' DDM details are shown: number of clusters per DDM, the average cluster size, and the classification accuracy of the best-performing cluster.

ID	zyg. stimulus			zyg. Speech			zyg. response			corr. stimulus			corr. speech			corr. response		
	# c	avg c size	max acc.	# c	avg c size	max acc.	# c	avg c size	max acc.	# c	avg c size	max acc.	# c	avg c size	max acc.	# c	avg c size	max acc.
02_03	0	0	0	3	12.6	81	0	0	0	1	19	60.5	2	5.5	62	2	13	65.5
02_04	10	42.3	69.5	1	22	61	3	13	70	1	5	65	4	10.2	66	1	5	63
03_05	3	19.3	72.5	1	41	72	5	17.8	69.5	0	0	0	5	19.6	79.5	1	32	69.5
03_06	1	16	64.5	3	10	63	2	18	64.5	1	6	65.5	1	44	64.5	1	15	60
04_09	2	75	67.5	0	0	0	3	124.3	74.5	0	0	0	3	19.3	68.5	0	0	0
04_10	0	0	0	2	18	71.5	0	0	0	1	10	75	0	0	0	0	0	0
05_13	1	7	60	2	17.5	59.5	0	0	0	3	13.3	60	0	0	0	2	11	58.5
05_14	5	38.2	76	2	0	0	2	21.5	70.5	0	0	0	0	20.5	66	0	0	0
06_15	7	12.1	63	0	0	0	8	31.1	76.5	0	0	0	7	18	64.5	1	8	59.5
07_18	2	7	63.5	1	12	60.5	1	11	62.5	3	10.3	66	3	6.3	64.5	2	10.5	60.5
08_19	1	9	65	0	0	0	0	0	0	1	61	67	3	22.6	83.5	0	0	0
08_20	10	19.1	66	7	21.5	66	4	14.2	64	0	0	0	5	26.8	65	1	15	63.5
09_27	0	0	0	0	0	0	2	99.5	68.5	0	0	0	9	31.8	73	3	15.3	66
09_28	11	16.4	63.5	2	15	70.5	6	33.1	68.5	0	0	0	4	12	61.5	0	0	0
10_07	2	21	67.5	6	0	0	0	42	73.5	0	0	0	0	0	0	0	0	0
10_08	7	50.7	77	3	8	68.5	7	44.4	82.5	5	7.2	69.5	2	30	74	2	20.5	63
11_22	1	22	64.5	0	0	0	0	0	0	0	0	0	0	0	0	0	0	0
12_23	0	0	0	3	128	82	0	0	0	6	49.1	78	0	0	0	5	37	74.5
12_24	0	0	0	0	0	0	0	0	0	3	24.3	66.5	0	0	0	1	46	61.5
13_25	3	5.6	67	1	6	60.5	10	15.6	65.5	0	0	0	3	9.3	64.5	1	7	61
14_11	0	0	0	4	46.7	67.5	0	0	0	4	45.2	70.5	1	12	69.5	2	26.5	76
14_12	0	0	0	8	30.6	71.5	0	0	0	6	32.3	70	1	5	68.5	0	0	0
15_29	4	10.5	61	7	0	0	8	12.1	63	0	0	0	0	10.5	62.5	0	0	0
15_30	4	17.7	67.5	0	0	0	7	10.7	65	1	12	64	3	7.6	63.5	1	7	66.5
16_31	3	7.3	64.5	4	9.7	70.5	4	9.75	65	2	15	64	3	9.6	64.5	1	5	73.5
16_32	3	6.6	61	9	61	77	2	6	61	4	53.2	74	1	9	58.5	0	0	0
17_33	4	8.7	62	4	11.2	64.5	5	15.6	63	1	8	60	3	9.6	64	10	17.3	65.5
18_37	2	10	60	0	0	0	3	13.6	60	2	10.5	59.5	4	36.7	63.5	11	36.1	63.5
18_38	1	28	63	4	20.2	65.5	0	0	0	1	10	62.5	0	0	0	1	7	63.5
19_39	2	8.5	62	2	11	59.5	3	10.3	59.5	0	0	0	4	10.5	63.5	1	6	59
19_40	3	14.3	64	0	0	0	7	21.2	67.5	0	0	0	8	18.6	67	1	17	67
20_41	3	7	61	0	0	0	6	11.8	82	0	0	0	5	9.8	61	0	0	0
21_43	1	8	59	6	29.1	72.5	2	11.5	60.5	2	129.5	76.5	0	0	0	0	0	0
22_45	0	0	0	2	7.5	60	1	10	60	3	6	64	1	12	61	7	38.2	67
22_46	3	7.6	67.5	4	0	0	4	12	67	0	0	0	0	22.7	63	0	0	0
23_47	0	0	0	0	0	0	1	14	82.5	0	0	0	1	9	58.5	1	8	57
23_48	1	9	59.5	0	0	0	4	10.5	78	0	0	0	3	10.6	58.5	1	8	58
24_49	2	88.5	67.5	2	7.5	72.5	1	228	75	1	27	66.5	1	10	59.5	0	0	0
24_50	7	11.7	66	0	0	0	4	12.5	65	0	0	0	5	14.2	65	3	9.6	74.5
25_52	0	0	0	0	0	0	1	0	0	0	0	0	0	15	81.5	0	0	0

c: cluster, acc: accuracy

Table S2. Classification results—Stage 2: with monetary incentives. Similar to Table S1, only in trials where participants were presented with monetary incentives for successful deception or detection.

ID	zyg. stimulus			zyg. speech			zyg. response			corr. stimulus			corr. speech			corr. response		
	# c	avg c size	max acc.	# c	avg c size	max acc.	# c	avg c size	max acc.	# c	avg c size	max acc.	# c	avg c size	max acc.	# c	avg c size	max acc.
02_03	0	0	0	2	16.5	64.5	0	0	0	2	15.5	69	0	0	0	3	24	63
02_04	2	24.5	68	3	20	64.5	8	18.1	71	10	13.5	66	9	23.8	66.5	4	33	65
03_05	4	22.5	68.5	0	0	0	3	31	81.5	0	0	0	0	0	0	1	16	66
03_06	2	70	64.5	4	11	61.5	4	67.5	70	0	0	0	0	0	0	0	0	0
04_09	3	145	70	0	0	0	7	35.4	71	0	0	0	4	34.7	72.5	0	0	0
04_10	0	0	0	2	67.5	71.5	0	0	0	1	14	70	0	0	0	4	21.5	66
05_13	1	12	57	6	23.6	62.5	0	0	0	7	17.2	61.5	0	0	0	8	13	62.5
05_14	0	0	0	2	0	0	0	10	73.5	0	0	0	0	0	0	0	0	0
06_15	5	13	62.5	0	0	0	2	18.5	60	0	0	0	3	35.3	91.5	2	9	58.5
07_18	8	10.5	69	1	7	62	4	20.5	65	0	0	0	7	11.5	64	0	0	0
08_19	6	24.5	75.5	7	15.2	68	9	19.2	77	0	0	0	2	130	85.5	1	48	72.5
08_20	8	17.2	65	3	12.3	65	11	20.2	67.5	5	12.8	64	6	14.6	81.5	2	14	63
09_27	5	17.8	49	6	80.3	80.5	1	21	68.5	3	28.6	72.5	2	63.5	70.5	3	9.6	68
09_28	3	12.6	73	0	0	0	7	12.8	61.5	0	0	0	3	12	62	0	0	0
10_07	0	0	0	7	0	0	4	20.4	74	0	0	0	0	91.25	72	0	0	0
10_08	3	7.6	70.5	4	13.2	69.5	4	24.5	72	10	11.8	72.5	3	15.3	68.5	2	196	74.5
11_22	2	12	63.5	6	33.8	78.5	2	15.5	76	1	150	93.5	1	5	62	3	33	72
12_23	0	0	0	1	31	70.5	2	5.5	62	4	11.5	67	0	0	0	0	0	0
12_24	2	16.5	60.5	1	30	63.5	3	6.6	63	1	37	68	3	13	63.5	2	26	65.5
13_25	10	20.2	68	2	23.5	62	16	15.3	70.5	1	27	66.5	14	17.5	67	1	21	62
14_11	0	0	0	3	18	68.5	1	10	63.5	4	35.5	71.5	2	8.5	60	3	19.3	72
14_12	0	0	0	6	19	68.5	4	20	64.5	3	16	64.5	1	16	62.5	6	10.6	74
15_29	6	15.5	65.5	12	0	0	4	15.5	65	0	0	0	0	21.7	87.2	0	0	0
15_30	17	18.4	70.5	7	17.5	73.5	6	25	68.5	4	41.7	71	5	19.4	68	3	64.6	73.5
16_31	7	16.4	68	6	72.5	79.5	14	21	71	2	18	71	6	36.8	73.5	4	70	79
16_32	0	0	0	3	45.3	66.5	0	0	0	2	52	67	0	0	0	1	27	63
17_33	8	21.8	65	1	8	59	7	24.8	66.5	1	6	61	7	16.4	63.5	1	7	60
18_37	7	12.4	64	4	7.2	63	1	6	61.5	4	73	63.5	6	17.8	63	0	0	0
18_38	2	6	61	0	0	0	1	8	60	0	0	0	2	6.5	60.5	2	9.5	60.5
19_39	8	26	67.5	1	11	59.5	7	15.8	64	7	10.7	63	4	13.5	65	6	7.1	62.5
19_40	5	17.6	65.5	1	43	67.5	6	24.8	69	0	0	0	4	17	69.5	0	0	0
20_41	8	24.3	70	0	0	0	13	19.6	69.5	1	8	59	9	44.4	74	2	6	64
21_43	0	0	0	0	0	0	2	6	61	1	20	68	1	18	59	3	6.6	68
22_45	10	21.2	65	7	20.8	67	8	11.6	63.5	8	9.8	65	2	14.5	61.5	6	45.1	68
22_46	4	10.7	63.5	8	0	0	7	9.5	65	0	0	0	0	9.8	64	0	0	0
23_47	0	0	0	3	13	63.5	0	0	0	3	9.6	62	3	11	67	11	22.2	66
23_48	4	9.5	61	2	10.5	60	7	12.1	88.2	1	5	60.5	6	11.1	65	0	0	0
24_49	1	59	67	2	11.5	59	1	189	65	0	0	0	1	23	63	2	9	64.5
24_50	4	17.5	85.8	1	12	67.5	7	16.5	68.5	2	10.5	70	4	17.2	69.5	1	7	63.5
25_52	1	9	78	2	0	0	0	10.5	59.5	0	0	0	0	0	0	0	0	0

c: cluster, acc: accuracy

Figure S1. An exemplary participant's sEMG signal during the calibration phase. The participant performed three voluntary smiling followed by three eyebrows contracting with a neutral expression in between (in response to a cued video). Each expression was performed for 3 s with a 3 s gap in between. Top 5 signals originate from electrodes placed over the *Zygomaticus major* (ZM) muscle (cheek region) and bottom 3 electrodes were placed over the *Corrugator supercilii* (CS) muscle (eyebrow region).

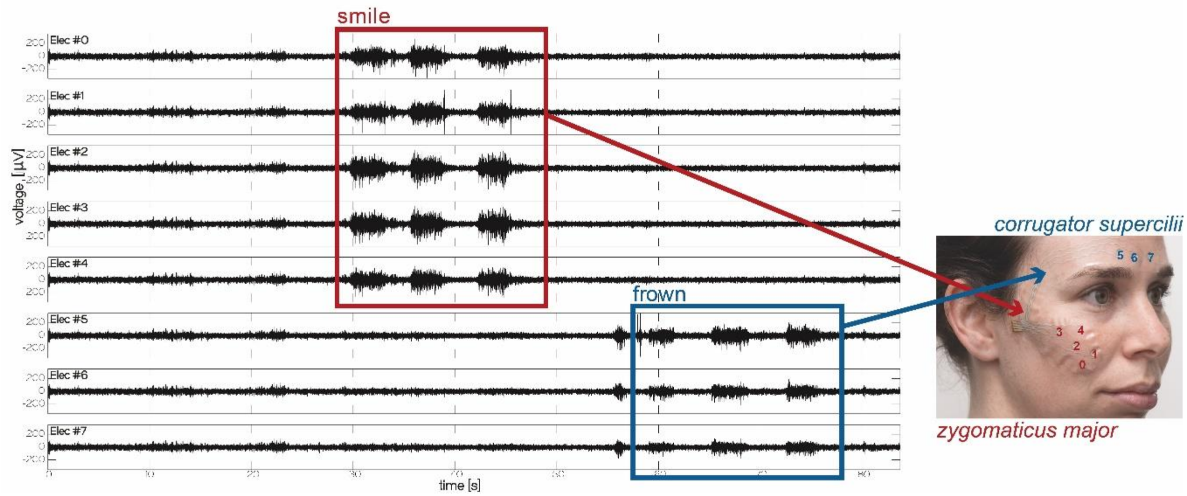

Figure S2. A flow chart of sEMG analysis stages. Starting with preprocessing, through signal selection, machine-learning classification analyses on the individual-participant level, and the aggregate multi-subject analyses.

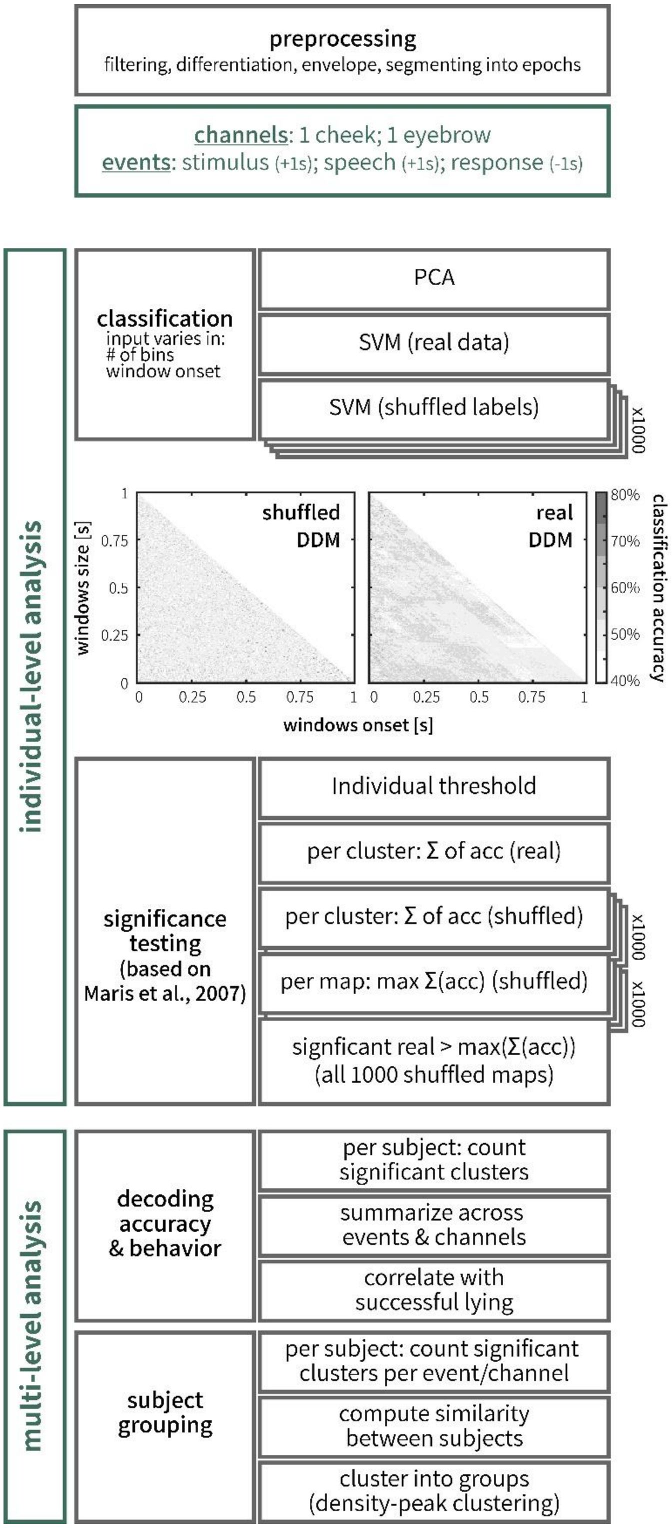

Figure S3. Illustration of the clustering procedure. Each row in the table and the similarity matrix represents a single participant. After calculating the similarity between each pair of participants, we generated a 'decision graph' where outliers represent cluster centroids. After selecting the centroids based on their gamma value (see Methods), the other participants were assigned to the cluster with the closest centroid. The similarity spaces are 2D projections of the 6-dimensional similarity space.

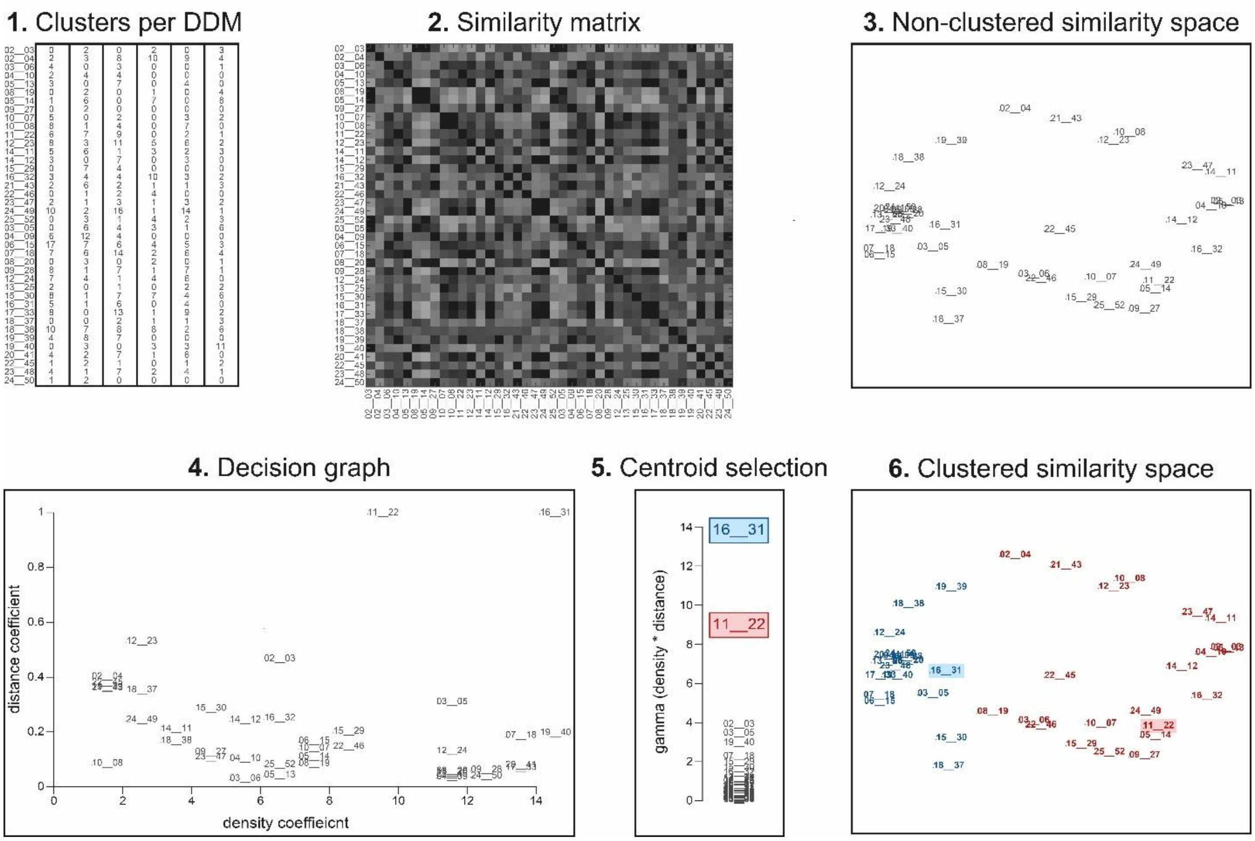

Supplement: Supplementary file 1 — Supporting Information [file BRB3-11-e2386-s001.pdf]
